# Enhanced Enzymatic Synthesis of Puerarin Palmitate with Different Acyl Donors for Lipid Solubility Improvement

**DOI:** 10.3390/ijms25020709

**Published:** 2024-01-05

**Authors:** Seungmee Lee, Hyeonmi Shin, Jihyun Bae, Taek Lee, Minji Kim, Heung Bae Jeon, Kang Hyun Lee, Hah Young Yoo, Chulhwan Park

**Affiliations:** 1 Department of Chemical Engineering, Kwangwoon University, Seoul 01897, Republic of Korea; 98lsm@naver.com (S.L.); lisames@naver.com (H.S.); bjh7778@naver.com (J.B.); tlee@kw.ac.kr (T.L.); 2 Department of Chemistry, Kwangwoon University, Seoul 01897, Republic of Korea; minji_iii@naver.com (M.K.); hbj@kw.ac.kr (H.B.J.); 3 Department of Bio-Convergence Engineering, Dongyang Mirae University, Seoul 08221, Republic of Korea; oys7158@dongyang.ac.kr; 4 Department of Biotechnology, Sangmyung University, Seoul 03016, Republic of Korea

**Keywords:** antioxidant, flavonoid, puerarin, flavonoid ester, lipase, lipid solubility

## Abstract

Puerarin is a flavonoid known as a natural antioxidant found in the root of *Pueraria robata*. Its antioxidant, anticancer, and anti-inflammatory effects have attracted attention as a potential functional ingredient in various bioindustries. However, puerarin has limited bioavailability owing to its low lipid solubility and stability. Acylation is proposed as a synthesis method to overcome this limitation. In this study, lipase-catalyzed acylation of puerarin and various acyl donors was performed, and the enzymatic synthetic condition was optimized. Under the condition (20 g/L of Novozym 435, palmitic anhydride, 1:15, 40 °C, tetrahydrofuran (THF)), the synthesis of puerarin ester achieved a significantly high conversion (98.97%) within a short time (3 h). The molecule of the synthesized puerarin palmitate was identified by various analyses such as liquid chromatography–mass spectrometry (LC–MS), Fourier-transform infrared spectroscopy (FT-IR), and carbon-13 nuclear magnetic resonance (^13^C NMR). The lipid solubility and the radical scavenging activity were also evaluated. Puerarin palmitate showed a slight decrease in antioxidant activity, but lipid solubility was significantly improved, improving bioavailability. The high conversion achieved for puerarin esters in this study will provide the foundation for industrial applications.

## 1. Introduction

Flavonoids, which are natural antioxidants, are secondary phenolic plant metabolites. They have attracted much attention because of their distinct pharmacological effects, such as antioxidant [1], anti-inflammatory [2], antidiabetic [3], and anticancer effects [4]. More than 15,000 flavonoids have been identified to date [5], and they are classified as flavanol, anthocyanidin, flavanone, flavonol, isoflavone, flavone, and chalcone [6]. Puerarin (daidzein-8-C-glucoside) is an isoflavone found primarily in the roots and bark of *Pueraria lobata*. Puerarin has treatment effects in coronary heart disease and vascular disease owing to its high antioxidant activity [7]. Moreover, it exerts anticancer effects by inhibiting cell proliferation and inducing apoptosis [8]. In addition, puerarin has been shown to be effective in treating alcoholism by alleviating liver damage and lipid accumulation caused by alcohol ingestion [9]. This demonstrates the effective preventive and therapeutic potential of puerarin.

Despite their wide range of bioactivities, flavonoids have limited bioavailability due to their low lipid solubility (lipophilicity) and stability [10,11,12]. Puerarin, a glycosylated flavonoid, has many hydroxyl groups in its molecular structure. As a result, puerarin is highly polar and hydrophilic, thereby reducing its solubility in lipids [13]. In addition, many hydroxyl groups cause puerarin to be eliminated from the body before it exerts its pharmacological effects. Due to its low lipid solubility and stability, its absorption by the human body is hindered, thereby limiting its application.

In this context, many studies have reported improvements in biological activity and bioavailability by derivatizing flavonoids. In particular, acylation has been suggested as a useful strategy in many studies [10,13,14,15,16]. Flavonoid esters synthesized via acylation have improved lipid solubility and, thus, increased absorption into the human body. Furthermore, due to its improved stability, it can exhibit therapeutic efficacy without being removed from the human body through protection from metabolic activity. Vaisali et al. studied the antioxidant effects of rutin and rutin fatty esters to determine the effects of acylation on oxidative stability [17]. Rutin ester with improved lipid solubility via acylation was more effective in preventing lipid oxidation than rutin. They reported that the improved lipid solubility of rutin esters resulted in more efficient interactions with colloidal substances. Similar to this study, Mo et al. synthesized puerarin ester via acylation and evaluated the in vivo absorption capacity of the target compound using the Caco-2 monolayer model [18]. According to these results, the absorption of puerarin octanoate in Caco-2 cells was approximately 2.69 times higher than that of puerarin, suggesting that puerarin esters have higher lipid solubility in the digestive system than puerarin. 

Acylation for the synthesis of flavonoid esters can be performed by chemical methods or enzymatic methods. However, acylation by chemical methods requires many protection and deprotection steps for the reactive hydroxyl groups of flavonoids. Low regioselectivity may also result in the formation of unwanted by-products, necessitating the inclusion of numerous purification processes for the removal of by-products and catalyst residues. The waste generated during this process can adversely affect the environment [19,20,21]. In contrast to chemical methods, enzymatic methods conduct the reaction under mild conditions, and the formation of by-products can be minimized due to high regioselectivity. As the production of by-products is reduced, fewer purification processes are required compared to chemical methods. As a result, an environmentally friendly process is feasible, as the waste is minimized. Therefore, many recent studies have been conducted on enzymatic reactions [10,13,22,23,24,25,26,27,28]. Various enzymes, such as transferases, isomerases, esterases, proteases, and lipases, can be used for acylation [21]. Among them, lipase can be used in various industrial processes, including the synthesis of food ingredients and biodiesel production [29]. In particular, immobilized lipase is mainly used for the enzymatic acylation of flavonoids [27]. Immobilized with lipase on an organic/inorganic support, the immobilized enzyme exhibits high operational stability and is easy to separate after the reaction, thereby reducing the difficulty in the purification process [10,13,23].

Lipase-mediated acylation can be affected by various variables (type of enzyme, concentration of enzyme, type of acyl donor, molar ratio of reactants, reaction temperature, and solvent). Therefore, it is important to determine the optimal synthetic conditions for obtaining a high conversion by investigating the influence of various variables. The one factor at a time (OFAT) method is one of the optimization strategies that can be used to investigate the effect of different variables on a response by changing one variable while holding other variables constant [10,13,19,22].

In this study, the effect of various variables (enzyme type, enzyme concentration, acyl donor, molar ratio, reaction temperature, and solvent type) on the synthesis of puerarin esters via enzymatic synthesis was investigated, and the optimal conditions were determined by the OFAT method. In particular, we focused on the synthesis of puerarin palmitate utilizing palmitic anhydride, methyl palmitate, vinyl palmitate, ethyl palmitate, and butyl palmitate as acyl donors, which is the first trial to our knowledge, and performed molecular identification and efficacy evaluation of the novel synthesized product. 

The identification of puerarin palmitate was achieved by analyzing the molecular structure and composition using Fourier-transform infrared spectroscopy (FT-IR) and carbon-13 nuclear magnetic resonance (^13^C NMR). 

## 2. Results and Discussion

### 2.1. Effect of Enzyme Type on the Conversion of Puerarin Ester

The selection of an appropriate enzyme type is essential for achieving optimal conversion in the enzymatic synthesis of puerarin ester. In this study, the reaction tendencies of three commercially available immobilized enzymes, namely Novozym 435, Lipozyme TL IM, and Lipozyme RM IM, were investigated. Novozym 435 is an enzyme that immobilizes *Candida antarctica* lipase B on an acrylic resin as a hydrophobic support. Lipozyme TL IM is an enzyme that immobilizes *Thermomyces lanuginosus* lipase on a cationic silicate support, while Lipozyme RM IM is an enzyme that immobilizes *Rhizomucor miehei* lipase on an anion exchange resin. The performance of three immobilized lipases was evaluated in a basal reaction system for conversion of puerarin ester, and 18.51%, 11.83%, and 16.17% conversions were achieved for Novozyme 435, Lipozyme TL IM, and Lipozyme RM IM, respectively. Based on these results, Novozym 435 showed a relatively higher conversion (Table 1) and was selected for the subsequent reactions.

The activity of an immobilized enzyme can be affected by the properties of the support material. In general, immobilized enzymes on hydrophobic supports exhibit higher activity compared to those on hydrophilic supports [22,30]. Since Novozym 435 is immobilized on an acrylic resin, which is a hydrophobic support, it demonstrated relatively high conversion in this study. Previous studies on the enzymatic synthesis of various flavonoids, including puerarin, have also reported a preference for Novozym 435 due to its superior activity [18]. In a similar study by Martins et al., where acylation was performed using various alcohols (ethanol, 2-propanol, 1-butanol, and 1-pentanol) and short-chain carboxylic acids (acetic acid, propionic acid, and butyric acid), Novozym 435 and Lipozyme RM IM showed higher conversion compared to Lipozyme TL IM [31], consistent with the findings of this study. These variations in conversion can be attributed to differences in lipase activity. Novozym 435 had four-fold higher activity than Lipozyme TL IM during synthesis, and Lipozyme TL IM had five-fold higher activity than Novozym 435 during hydrolysis. Lipozyme RM IM showed favorable activity in both synthesis and hydrolysis. As a result, Lipozyme TL IM gave the lowest yield due to its relatively low activity during synthesis, while Novozym 435 achieved relatively high yields, which is consistent with previous studies [19,22,23]. As a result, Novozym 435 was found to be an effective biocatalyst compared to other immobilized enzymes.

### 2.2. Effect of Enzyme Concentration on the Conversion of Puerarin Ester

**Figure 1:**
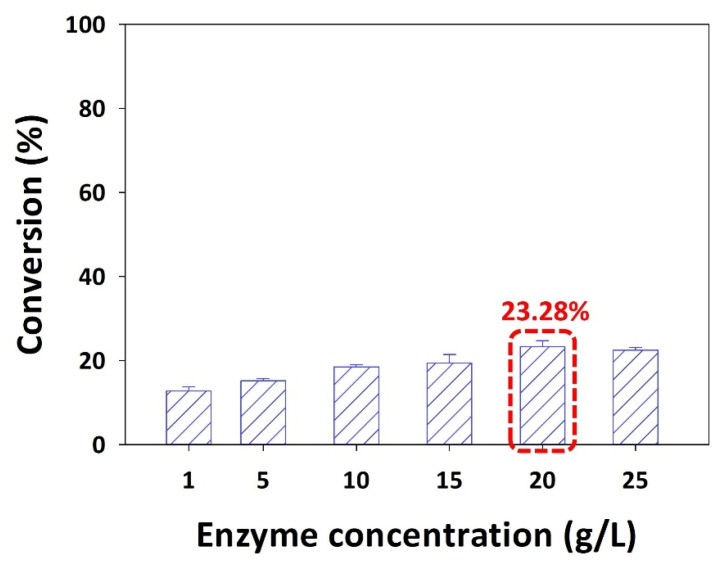
Effect of enzyme concentration on the conversion of puerarin ester (Novozym 435, palmitic acid as the acyl donor, 1:10 molar ratio of puerarin to acyl donor, reaction temperature of 40 °C, *tert*-amyl alcohol as the solvent, and reaction time of 48 h) (The optimal condition is indicated by a red box).

In an enzymatic reaction, the enzyme concentration plays a vital role in the conversion of substrate into a product. The enzyme concentrations were investigated in the range of 1–25 g/L. When 1 g/L Novozym 435 was used, the conversion was 12.79%. As the enzyme concentration increased to 5, 10, 15, and 20 g/L, the conversion gradually increased to 15.22%, 18.51%, 19.39%, and 23.28%, respectively. Meanwhile, when the enzyme concentration exceeded 25 g/L, the conversion decreased slightly to 22.45% (Figure 1). Therefore, 20 g/L was determined to be the optimal enzyme concentration for puerarin ester production.

The active site of the enzyme and substrate are bound together to form an enzyme–substrate complex in enzyme-based reactions. When the enzyme concentration in the reaction is increased, the active site of the enzyme is more exposed, and a large amount of enzyme–substrate complex is formed [22]. However, if the complex is formed excessively due to excessive addition of the enzyme, a steric hindrance effect on the enzyme active site occurs, preventing the binding of the enzyme and the substrate [10,13,23,30,32]. Another reason is that the hydrolysis reaction of the enzyme may affect the conversion to a product. Xiao et al. performed esterification of proanthocyanidin and palmitic acid using Novozym 435 and investigated the effect of enzyme concentration on synthesis [33]. As a result, the conversion increased as the enzyme concentration increased from 10 g/L to 40 g/L, but it was confirmed that the conversion slightly decreased at a concentration of 40 g/L or higher. This tendency has been attributed to the competition between the hydration and hydrolysis of enzymes. Similarly, in the study of acylation with naringin and decanoic acid using Novozym 435, it was determined that as the enzyme concentration was increased from 5 g/L to 20 g/L, the conversion increased [34]. This tendency was also observed during the enzymatic synthesis of octyl formate [19]. They investigated the concentration of Novozym 435 in the range of 5–30 g/L and reported that the conversion increased as the enzyme concentration increased but decreased once the concentration exceeded 15 g/L. Hence, it is important to use an appropriate amount of enzyme, and in this study, the optimal enzyme concentration was determined to be 20 g/L, which shows the maximum conversion.

### 2.3. Effects of Different acyl Donors on the Conversion of Puerarin Ester

Numerous studies have explored the synthesis of flavonoid esters using different types of acyl donors, including aromatic or aliphatic acids [13]. Among them, there has been a particular focus on fatty acids due to their ability to effectively improve lipid solubility (lipophilicity) [35]. In this section, acylation with fatty acids was performed to improve the lipid solubility of puerarin, and enzymatic reactions were performed with fatty acids of various chain lengths to select the acyl donor with the highest conversion. The acylation process involves either esterification or transesterification, depending on the type of acyl donor employed. The esterification of flavonoids with fatty acid generates water as a by-product, whereas the transesterification of flavonoids with fatty acid ester generates alcohol as a by-product. 

Initially, the effect of the chain length of acyl donor on puerarin ester conversion was investigated using fatty acids such as lauric acid, myristic acid, palmitic acid, stearic acid, and oleic acid. Esterification reactions between puerarin and fatty acids, excluding palmitic acid, resulted in conversions ranging from 13% to 16%, whereas palmitic acid exhibited a relatively high conversion rate of 23.28% (Table 2).

Previous studies have indicated that short-chain fatty acids have higher solvation capabilities compared to long-chain fatty acids [36,37]. This increased solvation effect can shift the reaction equilibrium towards hydrolysis. However, the use of long-chain fatty acids may not always be favorable for the synthesis reaction. In the case of Novozym 435, the optimal enzyme employed in our study, the active site features a narrow and deep funnel-shaped pocket structure [38,39,40]. Consequently, excessive chain length can lead to steric hindrance, impeding access to the enzyme’s active site [41]. Marcucci et al. reported the production of ethyl esters using fatty acids of different chain lengths found in oil and found that the highest yield was obtained with 16-carbon palmitic acid [42]. They proposed that solvation by short-chain fatty acids with fewer than 16 carbon atoms could result in hydrolysis. Moreover, if the carbon chain of the fatty acid exceeds 16, steric hindrance at the active site may hinder ester conversion. Therefore, based on its highest conversion rate, we selected palmitic acid as the fatty acid for subsequent reactions.

Subsequently, the effect of the type of acyl donor on the puerarin palmitate conversion was investigated using palmitic anhydride, methyl palmitate, vinyl palmitate, ethyl palmitate, and butyl palmitate. The results showed that reactions involving palmitic acid, methyl palmitate, ethyl palmitate, and butyl palmitate resulted in conversions below 30%. In contrast, vinyl palmitate and palmitic anhydride exhibited conversion rates of 50.46% and 55.52%, respectively, with palmitic anhydride showing a relatively high conversion rate (Table 3). 

Carboxylic acids, serving as acyl donors, are organic compounds containing a carboxyl group, and they can form various derivatives by substituting the hydroxyl group. These carboxylic acid derivatives act as acyl donors, providing an acyl group through nucleophilic acyl substitution reactions. The reactivity of nucleophilic acyl substitution reactions depends on the acyl group, with the reactivity order as follows: acid halide > acid anhydride > ester ≈ carboxylic acid > amide [10,43]. In this investigation, various compounds, including carboxylic acid, ester, and acid anhydride, were utilized as sources of acyl groups, with acid anhydride exhibiting the highest level of reactivity. Notably, palmitic anhydride, which contains two carbonyl groups, generated palmitic acid as a by-product during the synthesis of puerarin palmitate. This palmitic acid, in turn, could react with any remaining puerarin to produce puerarin palmitate. As a result, the use of palmitic anhydride led to relatively high conversion, establishing it as the preferred acyl donor for subsequent reactions. This trend aligns with previous findings in the synthesis of isoamyl acetate [44], where the introduction of acetic anhydride as an acyl donor facilitated a complex reaction involving two carbonyl groups. Similarly, a related study focusing on the synthesis of phenylacetate reported the highest conversion when acetic anhydride was used as the acyl donor [45]. Although previous several investigations have explored the effects of acetic anhydride as an acyl donor in flavonoid acylation [10], the utilization of palmitic anhydride had not been previously reported until this study, making it a novel approach in the field of flavonoid ester synthesis.

### 2.4. Impact of Molar Ratio on the Conversion of Puerarin Palmiate

**Figure 2:**
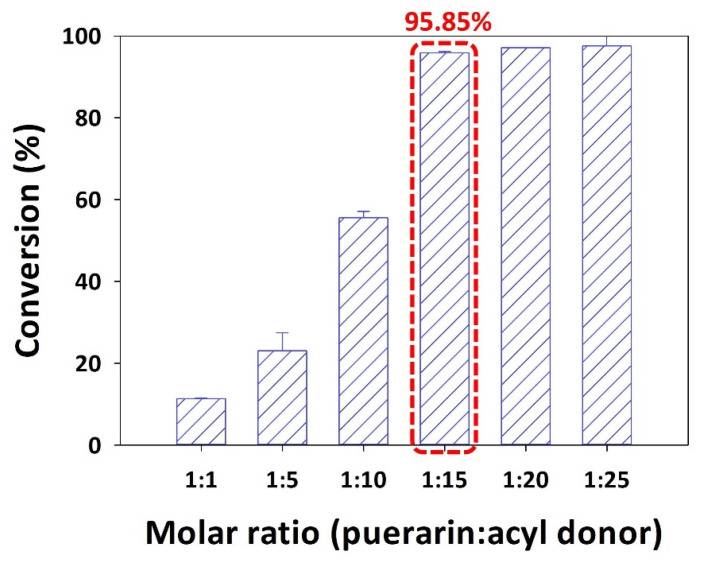
Effect of molar ratio of puerarin to acyl donor on the conversion of puerarin palmitate (20 g/L of Novozym 435, palmitic anhydride as the acyl donor, reaction temperature of 40 °C, *tert*-amyl alcohol as the solvent, and reaction time of 48 h) (The optimal condition is indicated by a red box).

To ensure a predominant forward reaction over hydrolysis, which is the reverse of acylation, an excess of acyl donor is crucial. In this study, the molar ratio of puerarin to palmitic anhydride was investigated, ranging from 1:1 to 1:25. Remarkably, as the molar ratio decreased from 1:1 to 1:15, a significant increase in conversion was observed, rising from 11.37% to 95.85%. However, further decreasing the molar ratio to 1:25 did not lead to a substantial increase in conversion (Figure 2). 

In general, the excess addition of reactants favors the thermodynamic equilibrium towards the synthesis direction in acylation reactions [10,13,22,30]. Nevertheless, depending on the mechanism of the lipase, an imbalance favoring an excess of puerarin rather than palmitic anhydride might hinder the smooth progression of the reaction. During the initiation of the enzymatic reaction, the active site of the enzyme combines with the acyl group of palmitic anhydride, forming a tetrahedral intermediate while releasing palmitic acid as a by-product. Subsequently, the hydroxyl group of puerarin, the acyl acceptor, binds to the active site of the enzyme, leading to the formation of a second tetrahedral intermediate. Ultimately, upon disintegration of the formed intermediate, a flavonoid ester is generated, and the enzyme’s active site returns to its original state. Consequently, promoting the binding between the lipase and acyl donor through excessive acyl donor addition can enhance the conversion rate. For instance, in a study investigating the acylation of puerarin and vinyl propionate in the presence of Novozym 435, decreasing the molar ratio of puerarin to vinyl propionate from 1:25 to 1:30 significantly increased the conversion [46]. However, subsequent increases in the concentration of vinyl propionate did not notably impact the conversion. Another study by Zheng et al. explored the effect of the molar ratio of flavonoid to α-linolenic acid on the synthesis of rutin linolenate and naringin linolenate using Novozym 435 [47]. Results from the evaluation spanning 1:1 to 1:8 (flavonoid:α-linolenic acid) indicated that as the molar ratio decreased, the conversion increased. However, once the molar ratio fell below a certain threshold, further decreases in the ratio had negligible effects on the conversion. The authors attributed this finding to the dependency of enzyme activity on the interaction between the substrate and the enzyme’s active site, wherein increased fatty acid concentrations influence the catalytic environment. 

### 2.5. Effect of Reaction Temperature on the Conversion of Puerarin Palmitate

**Figure 3:**
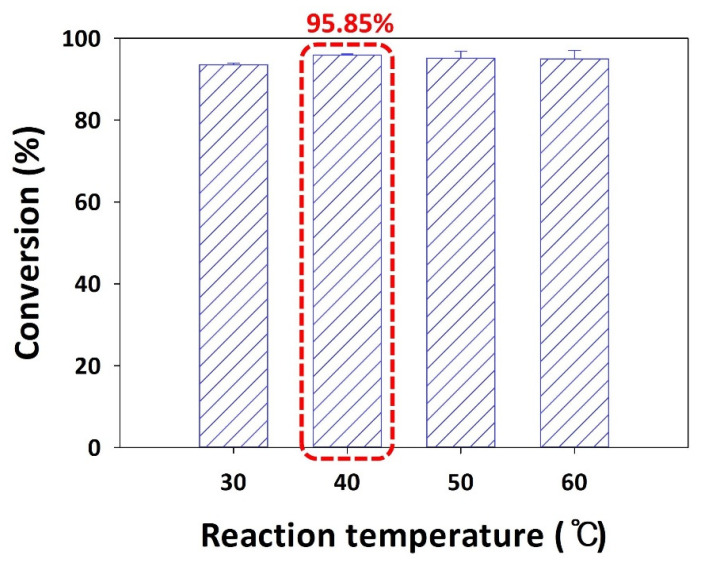
Effect of reaction temperature on the conversion of puerarin palmitate (20 g/L of Novozym 435, palmitic anhydride as the acyl donor, 1:15 molar ratio of puerarin to acyl donor, *tert*-amyl alcohol as the solvent, and reaction time of 48 h) (The optimal condition is indicated by a red box).

The determination of the most suitable reaction temperature is crucial due to its direct impact on enzyme activity. To determine the optimal temperature for puerarin palmitate synthesis, various reaction temperatures (30, 40, 50, and 60 °C) were investigated, considering the properties of solvents (freezing points and melting points), and the conversions were found to be 93.53%, 95.85%, 95.10%, and 94.90%, respectively (Figure 3).

The conversion of puerarin palmitate synthesis tended to increase as the temperature increased from 30 °C to 40 °C, but no significant results were observed after 40 °C. Therefore, the optimal reaction temperature for the highest conversion was determined to be 40 °C. In general, higher reaction temperatures lead to decreased mixture viscosity, thereby enhancing the reaction rate and promoting substrate–enzyme interaction. However, excessively high temperatures may result in enzyme denaturation and loss of activity [19,23]. Consistent with the findings of this study, previous research on the enzymatic synthesis of flavonoid esters has identified optimal reaction temperatures primarily within the range of 30–40 °C [32,48]. For instance, a study investigating esterification with flavonoids (phloridzin, isoquercitrin) using Novozym 435 evaluated temperature effects in the range of 40–70 °C [49]. The results indicated a rapid decrease in enzyme activity at temperatures above 60 °C, with optimal reaction rates observed between 40–45 °C. Similarly, another study focused on the esterification of naringin and oleic acid employing Lipozyme TL IM identified the optimal temperature as 40 °C, consistent with the findings of this study [13]. Furthermore, a recent study on transesterification of naringin with acetic anhydride or vinyl acetate using Lipozyme TL IM achieved the highest conversion at 40 °C [10].

### 2.6. Effect of Solvent on the Conversion of Puerarin Palmitate

The solvent used as a reaction medium is an important factor that greatly affects not only enzyme activity and stability but also substrate solubility. As the solubility of the substrate is greatly affected by the hydrophobicity of the solvent, the solvent was investigated based on the log *p* value, which is a measure of the hydrophobicity of the solvent. Therefore, it is important to identify a solvent that has a high solubility for both puerarin and palmitic anhydride and does not inhibit enzyme activity.

**Figure 4:**
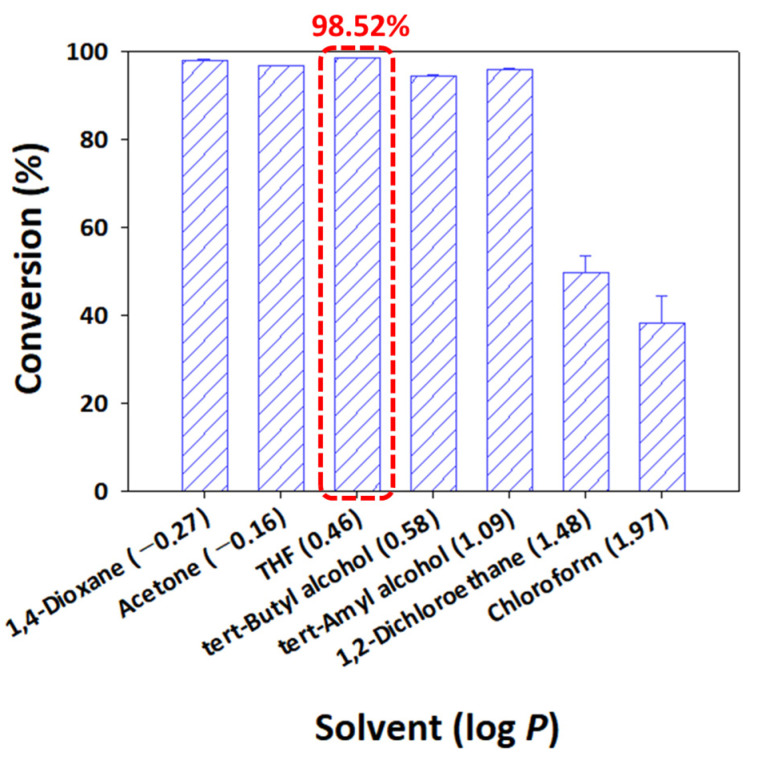
Effect of organic solvent on the conversion of puerarin palmitate (20 g/L of Novozym 435, palmitic anhydride as the acyl donor, 1:15 molar ratio of puerarin to acyl donor, reaction temperature of 40 °C, and reaction time of 48 h) (The optimal condition is indicated by a red box).

If the log *p* value was 2 or less, it was expressed as hydrophilic, and if it was 4 or more, it was expressed as hydrophobic [50]. When a hydrophobic solvent with a high log *p* value was used as the reaction solvent, hydrophilic puerarin did not dissolve. Conversely, when a strong hydrophilic solvent with an extremely low log *p* value was used, hydrophobic palmitic anhydride did not dissolve. Because the substrate does not dissolve in solvents, conversion into a product is not performed smoothly. Therefore, the reaction was conducted in a solvent with an appropriate log *p* value in which puerarin and palmitic anhydride are highly soluble. The solvents used in the reaction are as follows, in order of log *p* values: 1,4-dioxane (−0.27), acetone (−0.16), THF (0.46), *tert*-butyl alcohol (0.58), *tert*-amyl alcohol (1.09), 1,2-dichloroethane (1.48), and chloroform (1.97). When highly hydrophilic solvents, including 1,4-dioxane, acetone, THF, *tert*-butyl alcohol, and *tert*-amyl alcohol, were used, all showed high conversions of over 90%. However, the conversion gradually decreased as the reaction solvents became more hydrophobic, such as with 1,2-dichloroethane and chloroform. In particular, when 1,4-dioxane, acetone, and THF were used as solvents, the conversion rates consistently exceeded 95%. Notably, the use of THF as the reaction solvent yielded the highest conversion rate at 98.52 ± 0.01%, while 1,4-dioxane and acetone exhibited conversion rates of 98.05 ± 0.14% and 96.72 ± 0.07%, respectively (Figure 4). 

Using paired comparison analysis in OriginPro version 2024, which can work out the importance of a number of options relative to one another, we found that the conversion rate when acetone was used as a solvent was statistically different compared to the conversion rate when the other two solvents were used. The conversion rates when 1,4-dioxane and THF were used as solvents were statistically different, and THF, which showed a higher conversion rate, was selected as the optimal solvent.

An organic solvent with a too-low log *p* value and high polarity can inactivate the enzyme by removing the essential water layer from the active site of the enzyme [23]. Li et al., who conducted a study on the acylation of puerarin, reported that in a polar organic solvent with a log *p* value of −1.0 or less, the reaction did not proceed due to enzyme inactivation [51]. In addition, they confirmed that THF showed the highest efficiency for puerarin solubility and regioselectivity. Therefore, THF was chosen as the optimal reaction solvent because it has the highest conversion and can efficiently produce the target product.

### 2.7. Enzymatic Synthesis of Puerarin Palmitate under the Optimal Conditions

**Figure 5:**
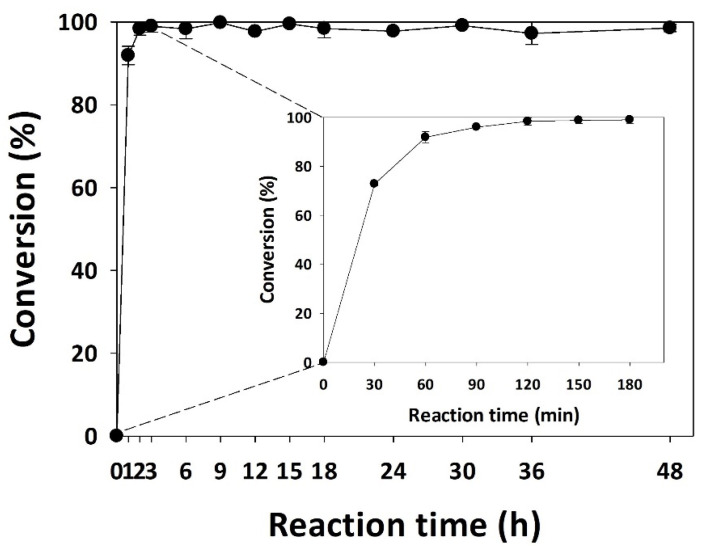
Conversion of puerarin palmitate as a function of reaction time (20 g/L of Novozym 435, palmitic anhydride as the acyl donor, 1:15 molar ratio puerarin to acyl donor, reaction temperature of 40 °C, and THF as the solvent).

The optimal conditions for the enzymatic synthesis of puerarin palmitate were determined, involving the use of Novozym 435 at a concentration of 20 g/L as the enzyme, palmitic anhydride as the acyl donor, a molar ratio of puerarin to acyl donor of 1:15, a reaction temperature of 40 °C, and tetrahydrofuran (THF) as the solvent. The conversion of the reaction was investigated at various time intervals ranging from 0 to 48 h, in particular at 0, 30, 60, 90, 120, 150, and 180 min. The conversion at these time points was 0%, 72.79%, 91.88%, 95.99%, 98.33%, 98.80%, and 98.97%, respectively (Figure 5). Remarkably, the synthesis of puerarin palmitate using lipase was accomplished within a short period of 3 h and maintained a stable conversion for the subsequent 48 h.

The significant reduction in reaction time can be attributed to the use of palmitic anhydride as the acyl donor. This acyl donor has two carbonyl groups, which facilitates the two-step reaction of puerarin palmitate synthesis. As a result, this study using palmitic anhydride as an acyl donor demonstrated a higher conversion in a shorter time compared to studies employing other acyl donors. This finding is consistent with our previous study optimizing the synthesis of naringin acetate, where it was observed that utilizing acetic anhydride led to a high conversion of 98.5% in a short time of 8 h [10].

Numerous studies have performed enzymatic synthesis of flavonoids and acyl donors (Table 4), and various attempts have been made to improve the reaction time and conversion for an efficient reaction. 

In general, studies using fatty acids as acyl donors require a long reaction time. Conversely, in the study using fatty acid esters as acyl donors, a high conversion can be achieved within a relatively short time. The synthesis reaction of puerarin and short-chain vinyl esters using Novozym 435 was reported to achieve a conversion of 98% within 6 h [18]. In addition, it was reported that the synthesis of naringin and acetic anhydride using Lipozyme TL IM achieved a conversion of 98.51% within 8 h [10]. In recent years, various attempts have been made to reduce the reaction time through additional processing into the reaction, in addition to changing the acyl donor. In particular, Guo et al. introduced an ultrasonic pretreatment process in the synthesis of naringin and palm oil to shorten the reaction time [52]. The conventional stirring method, which does not use ultrasound, required a reaction time of 48 h. However, the reaction time was reduced to 16 h by performing the ultrasonic pretreatment process for 30 min. In another study by Ziaullah and Ruasinghe, an ultrasonication technique to accelerate the reaction was suggested [49]. The acylation of phloridzin and isoquercitrin was performed by combining ultrasonic irradiation with a conventional stirring reaction. As a result, a similar conversion was achieved within 4.5 h, which was approximately five times faster than the conventional reaction time (24 h). In a separate study, they also suggested microwave-assisted enzymatic acylation using the same enzymes and flavonoids [53]. The conventional stirring method achieved a conversion of 82–98% through a reaction time of 18–24 h. In contrast, the microwave method completed the reaction within 120–160 s, which was significantly shorter than the conventional method. Based on these results, it was reported that acylation using ultrasound and microwave in an organic solvent using Novozym 435 provides a more efficient enzymatic synthesis method than the conventional stirring method. In this study, although no additional treatment was introduced, the reaction was completed within 3 h using palmitic anhydride as an acyl donor, and a high conversion of 98.97% was achieved. To the best of our knowledge, no study has been reported in which the reaction was completed in less than 3 h without the use of any additional treatments during the enzymatic reaction process. This was first achieved in this study. Moreover, the study findings indicate that the efficiency of the reaction can be improved if ultrasonic or microwave processing technology is applied in the subsequent research stages.

### 2.8. Analysis of Puerarin Palmitate

#### 2.8.1. High-Performance Liquid Chromatography (HPLC) and Liquid Chromatography–Mass Spectrometry (LC–MS) Analysis

**Figure 6:**
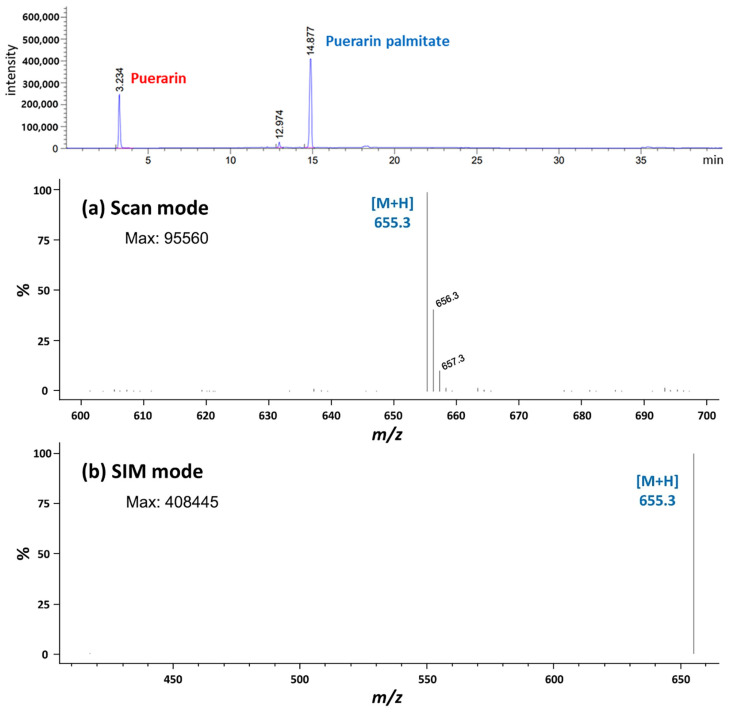
Mass spectra of puerarin palmitate in (**a**) scan mode and (**b**) SIM mode.

In this study, puerarin palmitate was synthesized via the acylation of puerarin and palmitic anhydride using lipase. Before the enzymatic reaction, only the peak of puerarin was observed using HPLC (Figure 6), with a retention time of 3.2 min. Puerarin palmitate was observed by HPLC peak as the reaction progressed, with an estimated retention time of 14.8 min. This peak, which was estimated to be puerarin palmitate, was confirmed by LC–MS analysis. Since the synthesized puerarin palmitate is an ester in which palmitic acid has bonded with puerarin and water has been released, it can be theoretically calculated that the molecular weight of puerarin palmitate is 654.8 g/mol, an increase of 256.4 g/mol and a decrease of 18.0 g/mol compared to the molecular weight of puerarin of 416.4 g/mol. Therefore, it was determined that puerarin palmitate had been synthesized.

#### 2.8.2. FT-IR and ^13^C NMR Analysis

**Figure 7:**
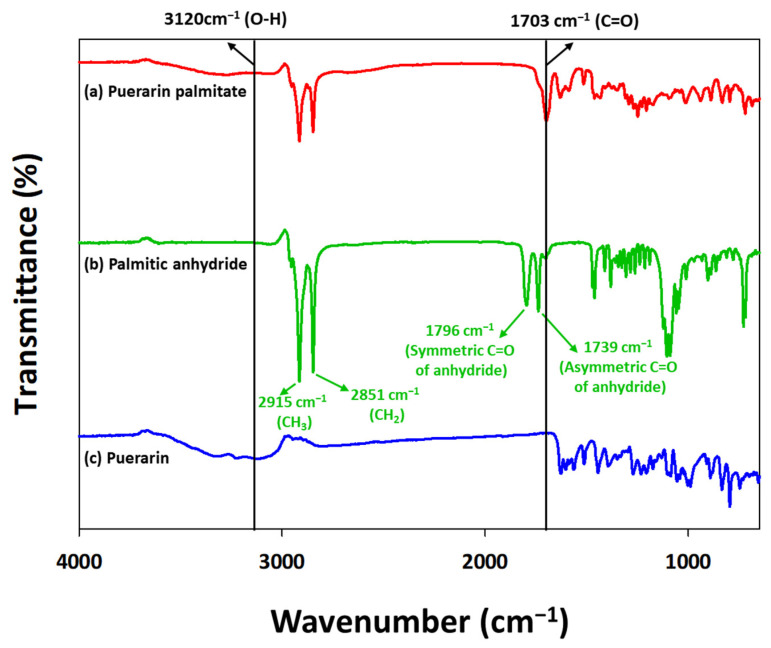
FT-IR spectra of (**a**) puerarin palmitate, (**b**) palmitic anhydride, and (**c**) puerarin.

FT-IR analysis was conducted on the reactants, puerarin and palmitic anhydride, and the purified product, puerarin palmitate (Figure 7). Consequently, the O-H stretch band (3120 cm^−1^) corresponding to the hydroxyl group of puerarin decreased in the product. Moreover, a C=O stretch band (1703 cm^−1^) belonging to the ester group was generated. This indicated that the hydroxyl group of puerarin and the carboxyl group of the acyl donor were combined via acylation to synthesize a puerarin ester with a carbonyl group. These results are consistent with those of other studies that synthesized various puerarin esters [51]. They reported that as a result of FT-IR analysis, a C=O stretch band (1710.80 cm^−1^) was observed in all puerarin esters. Studies on the synthesis of other flavonoid esters have reported similar results. Chebil et al. performed acylation of aglycone flavonoids (quercetin, naringenin, hesperetin, and chrysin) and determined whether the product was synthesized through FT-IR analysis [20]. They confirmed the production of flavonoid esters by observing a decrease in the O-H stretch band (3300 cm^−1^) of flavonoids and the appearance of the C=O stretch band (1720 cm^−1^) of flavonoid esters. In a study on the acylation of proanthocyanin dimer, the synthesis of the derivative was confirmed by the reduction of the O-H stretch band (3403 cm^−1^) and the generation of the C=O stretch band (1697 cm^−1^) [33]. Consequently, FT-IR analysis results indicated that puerarin palmitate was synthesized as a result of puerarin acylation.

**Figure 8:**
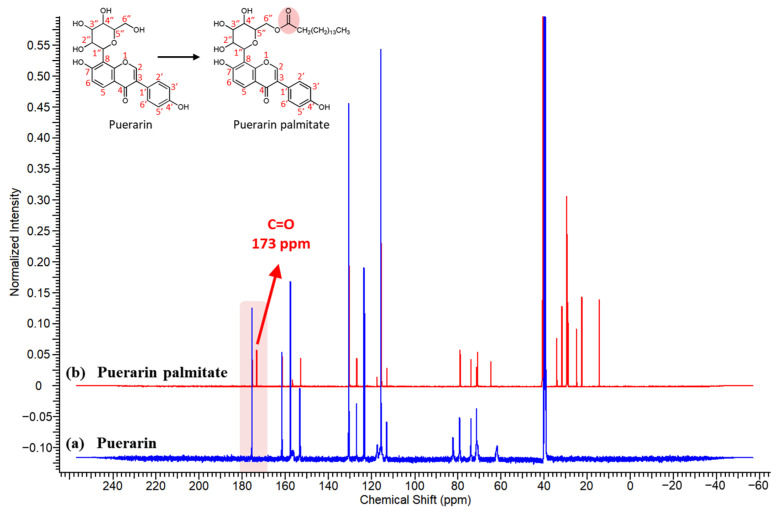
^13^C NMR spectra of (**a**) puerarin and (**b**) puerarin palmitate. (The newly created C=O double bond in puerarin palmitate after esterification is indicated by a red shade).

The changes in functional groups in the product were identified through ^13^C NMR analysis of the puerarin and the purified puerarin palmitate (Figure 8). As puerarin was converted to puerarin palmitate via acylation, the synthesized carbonyl group was observed at δ 173.42 ppm (C=O). A study on the synthesis of various puerarin esters revealed a carbonyl group observed at δ 174.16 ppm [51]. Furthermore, they reported that the acylation at the 6″-hydroxyl group causes a resonance effect on the carbonyl group of the ester. The C-6″ resonance shows a downfield shift, whereas the C-5″ resonance shows an upfield shift. Furthermore, the C-6″ resonance shifted from δ 61.86 ppm to δ 64.54 ppm, while the C-5″ resonance shifted from δ 82.26 ppm to δ 78.95 ppm. Similar chemical shifts were observed in this study. Thus, it was confirmed that the acylation was performed at the corresponding position and puerarin palmitate was successfully synthesized. Another study analyzing flavonoid esters with ^13^C NMR also confirmed the formation of carbonyl groups at a similar position. Razak et al. synthesized rutin laurate via acylation with rutin and lauric acid, and ^13^C NMR analysis confirmed the presence of a peak related to the carbonyl group of the ester at 173.2 ppm [54]. In addition, in the study on the synthesis of esculin palmitate and rutin palmitate, the presence of the carbonyl group of the ester at the position of 173.5 ppm was also confirmed through ^13^C NMR analysis [55].

#### 2.8.3. Evaluation of Lipid Solubility and Antioxidant Activity of Puerarin Palmitate

The lipid solubility of puerarin palmitate was evaluated by determining the log *p* value. The value of log *p* is closely related to the absorption and metabolism of compounds into the body. Therefore, it is important to determine an appropriate log *p* value for application to a living body. Few studies on the synthesis of flavonoid esters determined the log *p* values to evaluate the improvement in lipid solubility of the synthesized materials. In this study, log *p* was determined using the 1-octanol/water partition coefficient. The log *p* values of puerarin and puerarin palmitate were −0.22 and 3.49, respectively (Table 5). The log *p* value of puerarin, which was negative before synthesis, rapidly increased to a positive value after acylation. Hence, it was confirmed that the lipid solubility of puerarin palmitate was significantly improved compared with that of puerarin. Similar to this study, in the study of puerarin synthesis with different acyl donors, lipid solubility was evaluated by determining log *p* values for all derivatives [51]. All synthesized puerarin esters showed higher lipid solubility than puerarin. The log *p* value of puerarin was approximately −0.5, which is similar to the value obtained in our study. Furthermore, according to a study evaluating the lipophilicity of various naringin esters, the log *p* value of naringin was significantly improved via acylation [52]. They reported that the log *p* of naringin esters increased as the chain length of the fatty acids increased. Many previous studies have investigated the acylation of flavonoids using fatty acids to improve their low lipid solubility [13,32,56]. Moreover, several studies have evaluated the lipid solubility of flavonoid esters [29,33,36]. The lipid solubility of puerarin palmitate was evaluated for the first time. In addition, a higher improvement was achieved by using a longer-chain acyl donor than in previously reported studies on puerarin esters.

**Figure 9:**
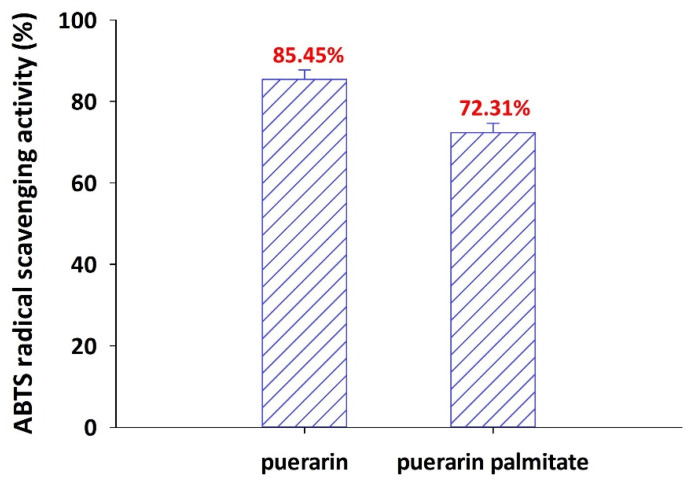
Antioxidant activity of puerarin and puerarin palmitate using ABTS assay.

The antioxidant of puerarin palmitate was evaluated using 2,2′-azino-bis(3-ethylbenzothiazoline-6-sulfonic acid ammonium salt) (ABTS) assay. Flavonoids, including puerarin, are expected to be utilized in various industries owing to their excellent antioxidant activity. The synthesis of puerarin ester via acylation allows puerarin to be absorbed into the body and exert pharmacological effects. Therefore, it is important to determine whether acylation, which is used to improve lipid solubility, reduces antioxidant activity. In this study, the antioxidant activity of puerarin palmitate was confirmed using the ABTS assay. As puerarin was converted to puerarin palmitate, the antioxidant activity decreased slightly, but was maintained at over 70% (Figure 9). 

In a previous study that synthesized various puerarin esters and evaluated their antioxidant activity, puerarin ester showed lower antioxidant capacity than puerarin [51]. This decrease in activity was attributed to the steric effect caused by the modification of the phenolic hydroxyl group, as well as the effects of the lipophilic end in the acyl donor. The lipophilic ends generated by acylation agglomerate with each other to reduce the interaction with free radicals, thereby reducing the free radical scavenging ability. In a study evaluating the antioxidant activity of naringin and naringin ester with the 2,2-diphenyl-1-picrylhydrazyl (DPPH) assay, the free radical scavenging ability of naringin ester was reduced; however, similar to this study, all derivatives maintained more than 70% activity [29]. In our study, the newly synthesized puerarin palmitate using long-chain fatty acids longer than C12 showed a slight decrease in antioxidant activity compared to puerarin (control), but significantly improved lipid solubility, which is expected to improve bioavailability by increasing absorption in the body. In particular, this is the first successful synthesis of puerarin palmitate by using anhydrous palmitic acid as an acyl donor, and it could become a prominent practice to overcome the limitations of natural ingredients in the pharmaceutical, cosmetic, and food industries. However, the synthesis was performed only at the laboratory scale and has limitations for economic analysis; thus, further studies on process demonstration and its reproducibility at the bench or pilot scale are required. In particular, continuous reactions should be considered for industrial applications to improve productivity and economics by reducing the amount of equipment and downstream processes [59]. Therefore, various applied research studies will be carried out in our follow-up work in the near future, including the development of a continuous reaction system, statistical optimization of puerarin esterification with palmitic anhydride, and economic analysis through process simulation.

## 3. Materials and Methods

### 3.1. Materials

Puerarin was purchased from Alibaba Co., Ltd. (Hangzhou, China). Lauric acid, tetrahydrofuran (THF), and acetonitrile were purchased from Junsei (Tokyo, Japan), while myristic acid, palmitic acid, stearic acid, oleic acid, 1,4-dioxane, acetone, *tert*-butyl alcohol, *tert*-amyl alcohol, 1,2-dichloroethane, chloroform, and n-heptane were purchased from Dae-Jung (Sigeung-si, Republic of Korea). In addition, palmitic anhydride, vinyl palmitate, ethyl palmitate, butyl palmitate, 1-octanol, and ABTS were obtained from TCI (Tokyo, Japan), and methyl palmitate was purchased from Aladdin (Shanghai, China). Ethyl acetate and dimethyl sulfoxide-*d*_6_ (DMSO-*d*_6_) were purchased from Sigma-Aldrich (St. Louis, MO, USA). Commercially used immobilized enzymes, Novozym 435 (*Candida antarctica* lipase B immobilized on acrylic resin), Lipozyme TL IM (*Thermomyces lanuginosus* immobilized on a silica gel carrier), and Lipozyme RM IM (*Rhizomucor miehei* immobilized on a resin carrier) were obtained from Novozymes (Bagsværd, Demark). The acetic acid, acetonitrile, methanol, and water used for HPLC analysis were purchased from J.T.Baker (Phillipsburg, NJ, USA) in HPLC grade. Formic acid used for LC–MS analysis was purchased from Thermo Fisher Scientific (Waltham, MA, USA).

### 3.2. Enzymatic Synthesis of Puerarin Ester

**Figure 10:**
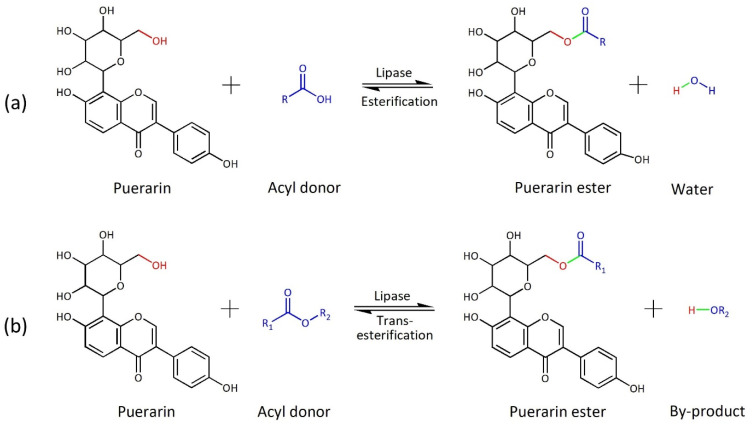
Reaction mechanism for the production of puerarin ester from puerarin and acyl donors via (**a**) esterification and (**b**) transesterification (The functional group of puerarin is shown in red, the functional group of the acyl donor is shown in blue, and the functional group newly created due to the reaction is shown in green).

Puerarin ester was synthesized via enzymatic synthesis using lipase (Figure 10). The enzymatic synthesis of puerarin ester was performed using a shaking incubator while maintaining a working volume of 10 mL in a 50 mL serum bottle. Puerarin (10 mM) and the acyl donor (10–250 mM) were dissolved in an organic solvent and placed in a serum bottle. A certain concentration of enzyme (1–25 g/L) was then added. A serum bottle containing the enzyme-containing reaction solution was sealed to prevent evaporation. The reaction was then performed at the same temperature at 180 rpm for 48 h in a shaking incubator. Following the reaction, 100 μL of the reaction solution was collected, diluted 10 times with methanol, filtered, and analyzed by HPLC. All experiments were repeated at least in triplicate, and the data are presented as the mean ± standard deviation of the measured or calculated values.

### 3.3. Optimization of Reaction Conditions

**Figure 11:**
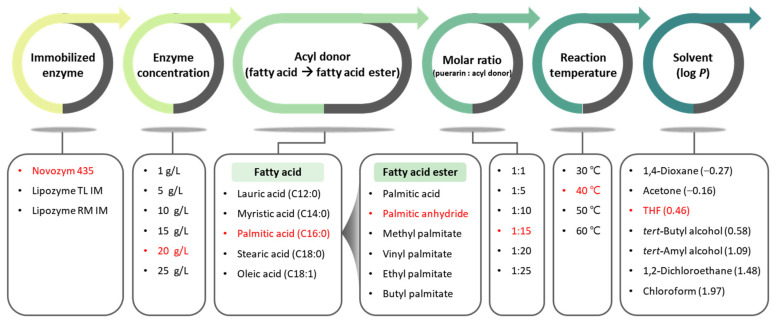
Optimization procedure for puerarin palmitate synthesis (Optimal conditions are indicated in red).

Reaction conditions for the synthesis of puerarin ester were optimized step-by-step using the OFAT method for six variables that affect the reaction: enzyme type, enzyme concentration, acyl donor type, molar ratio of reactants, reaction temperature, and solvent (Figure 11). The reaction was optimized based on the basic reaction conditions (10 g/L of Novozym 435, palmitic acid as an acyl donor, molar ratio of 1:10, reaction temperature of 40 °C, and *tert*-amyl alcohol as the solvent). All the reactions were conducted in a shaking incubator at 180 rpm for 48 h.

First, the effect of the type of enzyme on the reaction was determined using three commercially available immobilized enzymes (Novozym 435, Lipozyme TL IM, and Lipozyme RM IM). Each enzyme was used at a concentration of 10 g/L, and palmitic acid was used as an acyl donor. In addition, the molar ratio of puerarin to palmitic acid was 1:10, the reaction temperature was maintained at 40 °C, and *tert*-amyl alcohol was used as the reaction solvent.

Following the determination of the optimal enzyme (Novozym 435), the effect of enzyme concentration was investigated at concentrations of 1, 5, 10, 15, 20, and 25 g/L. Palmitic acid was used as the acyl donor, and the molar ratio of the reactants was 1:10. At a reaction temperature of 40 °C, *tert*-amyl alcohol was used as a solvent.

The subsequent synthesis reaction was performed using various acyl donors at a determined concentration of the enzyme (Novozym 435, 20 g/L). The optimal fatty acid was determined by evaluating the effects of various chain lengths (lauric acid, myristic acid, palmitic acid, stearic acid, and oleic acid). Based on the determined fatty acid, the optimal acyl donor was determined by investigating the effects of various fatty acid esters (palmitic anhydride, methyl palmitate, vinyl palmitate, ethyl palmitate, and butyl palmitate), including the selected fatty acid (palmitic acid). The molar ratio of reactants was set at 1:10, the reaction temperature was maintained at 40 °C, and *tert*-amyl alcohol was used as the reaction solvent.

The optimal molar ratio was determined by adjusting the molar ratios of the reactants (1:1, 1:5, 1:10, 1:15, 1:20, and 1:25) using the acyl donor (palmitic anhydride). In the presence of Novozym 435 (20 g/L), *tert*-amyl alcohol was used as the solvent at a reaction temperature of 40 °C.

The reaction temperature was investigated over the ranges 30, 40, 50, and 60 °C using the determined molar ratio (1:15). Novozym 435 (20 g/L) was used as the enzyme, palmitic anhydride as the acyl donor, and *tert*-amyl alcohol as the reaction solvent.

Finally, the optimal reaction conditions were evaluated by investigating various organic solvents (1,4-dioxane, acetone, THF, *tert*-butyl alcohol, *tert*-amyl alcohol, 1,2-dichloroethane, and chloroform) at the determined temperature (40 °C). In the reaction, 20 g/L Novozym 435 was used as the enzyme, and palmitic anhydride was used as an acyl donor. The reaction was carried out at a molar ratio of 1:15.

### 3.4. Purification of Puerarin Palmitate

The obtained product, puerarin palmitate, was purified by liquid–liquid extraction. The immobilized enzyme in the reaction mixture that reacted under optimal conditions was isolated through filtration, and the reaction solution was dried using a vacuum desiccator. The powder obtained by drying was dissolved in 35 mL of an acetonitrile/n-heptane (2:5, *v*/*v*) solution at 60 °C. The unreacted acyl donor in the n-heptane phase was then removed by separating the acetonitrile phase. The separated acetonitrile phase was then dried in a vacuum desiccator. To remove unreacted puerarin, the obtained powder was dissolved in 40 mL of an ethyl acetate/water (3:5, *v*/*v*) solution at 60 °C. Puerarin palmitate was finally obtained by separating and drying the ethyl acetate phase in which the puerarin ester had been dissolved. All the processes were repeated three times to improve the purity of the product. Afterwards, it was purified using thin-layer chromatography (TLC) and column chromatography on silica to obtain a higher-purity product. A mixture of ethyl acetate:chloroform:methanol (5:4:1, *v*/*v*/*v*) was used as the eluent.

### 3.5. Analysis of Puerarin Palmitate

#### 3.5.1. HPLC Analysis

The synthesis of puerarin ester was monitored by HPLC analysis using an Agilent 1260 Infinity II (Agilent Technologies, Santa Clara, CA, USA). Following the completion of the reaction, 100 μL of the reaction mixture was diluted 10-fold with methanol. The diluted solution was injected into a 2 mL vial and then analyzed after removing the remaining enzyme and residue using a syringe filter. 

Analysis was performed using an INNO Column C18 (120 Å, 5 μm, 4.6 mm × 250 mm) with a 5 μL injection volume and a column temperature of 50 °C. The mobile phases used were 1% acetic acid in water (A) and methanol (B). For analysis, the flow rate of the mobile phase was maintained at 1 mL/min, and the gradients were as follows: (A/B) 0 min-70/30, 5 min-0/100, 10 min-0/100, 15 min-70/30, and 20 min-70/30. The substance was detected by UV spectroscopy at 250 nm. The conversion of puerarin to its ester was calculated by dividing the concentration of reacted puerarin by the initial concentration of puerarin, as shown in Equation (1).

(1) $$\mathrm{Conversion}\;\left(\%\right)=\frac{{\left[C\right]}_{\mathrm{Puerarin},\;\mathrm{initial}}-{\left[C\right]}_{\mathrm{Puerarin},\;\mathrm{residual}}}{{\left[C\right]}_{\mathrm{Puerarin},\;\mathrm{initial}}}\times 100$$

#### 3.5.2. LC–MS Analysis

LC–MS was performed using Agilent 1260 Infinity II and Infinity Lab LC/MSD (Agilent Technologies). The type of column used in the analysis, the injection volume, and the column temperature were all maintained the same as for the HPLC analysis. For the mobile phase, 0.01% formic acid in water (A) and 0.01% formic acid in acetonitrile (B) were used for analysis at a flow rate of 1 mL/min, with the following gradient: (A/B) 0 min-70/30, 10 min-15/85, 20 min-15/85, 25 min-0/100, 30 min-0/100, 35 min-70/30, 40 min-70/30. Electrospray ionization (ESI) was used in the positive ion mode. In addition, the drying gas flow was set to 12 L/min, the drying gas temperature was set to 350 °C, the capillary voltage was set to 4000 V, and the nebulizer pressure was set to 35 psi. The material was detected at 250 nm using a diode array detector (DAD). Analysis of puerarin palmitate was performed in scan mode (*m*/*z* 600–700) and selected ion monitoring (SIM) mode (*m*/*z* 655.3).

#### 3.5.3. FT-IR and ^13^C NMR Analysis

The presence and changes in the main binding of purified puerarin palmitate were confirmed by FT-IR analysis and ^13^C NMR analysis. The FT-IR spectra were recorded in the frequency range 650–4000 cm^−1^ using an FT-IR spectrometer (JASCO FT/IR-4600, Tokyo, Japan). ^13^C NMR spectra were recorded using an NMR spectrometer (Oxford NMR AS400, Oxford, UK) at 400 MHz, and the chemical shifts were indicated in ppm. For ^13^C NMR analysis, puerarin and puerarin palmitate were dissolved in DMSO-*d*_6_ and then analyzed.

### 3.6. Evaluation of Lipid Solubility and Antioxidant Activity of Puerarin Palmitate

#### 3.6.1. Evaluation of Lipid Solubility of Puerarin Palmitate

The improved lipid solubility (lipophilicity) of puerarin palmitate was evaluated by measuring the log *p* value, which is a general method for determining the lipid solubility [52]. To determine the log *p* value of the 1-octanol/water partition coefficient, a solution of 1-octanol and water was saturated at 40 °C for 24 h. Following that, the sample was added, mixed in a shaking incubator at 40 °C for 24 h, and then left still for 24 h. Each solution in the 1-octanol phase and water phase was collected, filtered, and analyzed by HPLC. Finally, the log *p* value was determined using Equation (2).

(2) $$\log\;p=\log\;\left(\frac{{\left[C\right]}_{octanol}}{{\left[C\right]}_{water}}\right)$$

#### 3.6.2. Evaluation of Antioxidant Activity of Puerarin Palmitate Using ABTS Assay

The antioxidant activities of puerarin and puerarin palmitate were evaluated based on their ability to remove ABTS radical cations. First, to evaluate ABTS radical scavenging activity, an ABTS radical cation solution was prepared by mixing 7 mM ABTS solution with 2.45 mM potassium persulfate solution in a 1:1 ratio. This solution was diluted with methanol, and the absorbance was set to 1.1 (±0.02) at 734 nm using a UV/Vis spectrophotometer. Following that, 950 μL of ABTS radical cation solution was prepared as a control solution mixed with 50 μL of methanol and a sample solution mixed with 50 μL of diluted samples (puerarin and puerarin palmitate).

The absorbance of the prepared control and sample solutions was measured after 30 min of reaction at 25 °C. Samples were prepared at 1 mM, and all experiments were independently repeated three times. The radical scavenging capacity for ABTS was calculated according to Equation (3).

(3) $$\mathrm{radical}\;\mathrm{scavenging}\;\mathrm{activity}\;\left(\%\right)=\left(1-\frac{sample\;OD}{control\;OD}\right)\times 100$$

## 4. Conclusions

In this study, the enzymatic synthesis of puerarin palmitate was conducted to enhance the limited lipid solubility of puerarin, and the major variables influencing the synthesis were optimized. We set six major variables, including enzyme type, enzyme concentration, acyl donor type, and molar ratio of reactants, which are commonly considered in the enzymatic synthesis of flavonoid esters. Through the OFAT method, a remarkable conversion rate of 98.97% was achieved within 3 h under optimal conditions: 20 g/L of Novozym 435, palmitic anhydride, with a 1:15 molar ratio of puerarin to palmitic anhydride, at 40 °C in THF. The successful synthesis of puerarin palmitate was confirmed by HPLC and LC–MS, and its structural characteristics were analyzed by FT-IR and ^13^C NMR. In addition, the antioxidant activity of puerarin palmitate was evaluated using the ABTS assay, while its lipid solubility was determined by the log *p* value. Although the antioxidant activity of puerarin palmitate slightly decreased compared to that of puerarin, it remained above 70%. In particular, the synthesis significantly enhanced the lipid solubility, transforming it from negative to positive, thereby positively impacting its bioavailability. To the best of our knowledge, this is the first report on the utilization of palmitic anhydride as an acyl donor for flavonoid acylation. In addition, while previous studies have explored puerarin ester synthesis through acylation, the synthesis of puerarin palmitate has remained unexplored until now. Consequently, this study successfully synthesized puerarin palmitate under optimized conditions, marking a significant breakthrough in the field. The incorporation of palmitic anhydride as the acyl donor in the synthesis of flavonoid esters represents a novel contribution, addressing limitations and opening up potential applications in the pharmaceutical, cosmetic, and food industries. 

## Figures and Tables

**Table 1:** Summary of immobilized lipase and the effect of enzyme type on the conversion of puerarin ester (10 g/L of the enzyme, palmitic acid as the acyl donor, 1:10 molar ratio of puerarin to acyl donor, reaction temperature of 40 °C, *tert*-amyl alcohol as the solvent, and reaction time of 48 h).

Immobilized Lipase (Regioselectivity)	Source	Support	Conversion (%)
Novozym 435 (nonspecific)	*Candida antarctica* lipase B	Acrylic resin	18.51 ± 0.01
Lipozyme TL IM (1,3-specific)	*Thermomyces* *lanuginosus*	Silica resin	11.83 ± 0.17
Lipozyme RM IM (1,3-specific)	*Rhizomucor* *miehei*	Anion-exchange resin	16.17 ± 0.52

**Table 2:** Information on acyl donors and the effect of acyl donor type via esterification (20 g/L of the Novozym 435, 1:10 molar ratio puerarin to acyl donor, reaction temperature of 40 °C, tert-amyl alcohol as the solvent, and reaction time of 48 h).

Acyl Donor	Molecular Formula	Structure	Molecular Weight (g/mol)	Conversion (%)
Lauric acid	C_12_H_24_O_2_	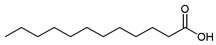	200.32	13.73 ± 1.66
Myristic acid	C_14_H_28_O_2_	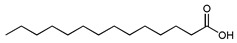	228.37	13.66 ± 0.10
Palmitic acid	C_16_H_32_O_2_	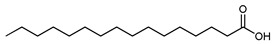	256.40	23.28 ± 0.07
Stearic acid	C_18_H_36_O_2_	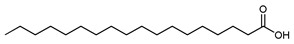	284.48	15.38 ± 0.62
Oleic acid	C_18_H_34_O_2_	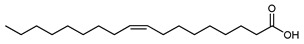	282.47	13.04 ± 0.57

**Table 3:** Information on acyl donors and the effect of acyl donor type via transesterification (20 g/L of Novozym 435, 1:10 molar ratio of puerarin to acyl donor, reaction temperature of 40 °C, *tert*-amyl alcohol as the solvent, and reaction time of 48 h).

Acyl Donor	Molecular Formula	Structure	Molecular Weight (g/mol)	Conversion (%)
Palmitic acid	C_16_H_32_O_2_	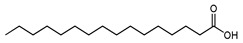	256.40	23.28 ± 0.07
Palmitic anhydride	C_32_H_62_O_3_	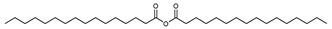	494.85	55.52 ± 1.11
Methyl palmitate	C_17_H_34_O_2_	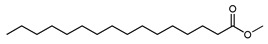	270.45	16.76 ± 0.09
Vinyl palmitate	C_18_H_34_O_2_	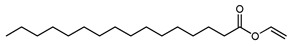	282.50	50.46 ± 0.69
Ethyl palmitate	C_18_H_36_O_2_	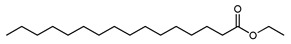	284.48	19.74 ± 0.84
Butyl palmitate	C_20_H_40_O_2_	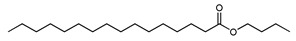	312.54	19.46 ± 0.96

**Table 4:** Summary of reaction conditions for the enzymatic synthesis of flavonoid esters.

Flavonoid	Acyl Donor	Enzyme, Enzyme Conc.	Molar Ratio (Flavonoid: Acyl Donor)	Reaction Temp.	Solvent	Reaction Time (Shortened Time), Conversion	Ref.
Naringin	Oleic acid	Lipozyme TL IM, 10 g/L	1:20	40 °C	Acetonitrile	24 h, 92.17% 48 h, 93.10%	[13]
Naringin	Acetic anhydride, vinyl acetate	Lipozyme TL IM, 3 g/L	1:5	40 °C	Acetonitrile	8 h, 98.51%, 24 h, 97.54%	[10]
Naringin	Palm oil	Lipozyme TL IM, 50 g/L	1:10	55 °C	Acetone	48 h (16 h), 93.90%	[52]
Isoquercitrin phloridzin	Fatty acids (C18–C22)	Novozym 435, -	-	45–60 °C	Acetone	18–24 h (120–160 s), 82–98%	[53]
Proanthocyanidin	Palmitic acid	Novozym 435, 30 g/L	1:10	60 °C	*tert*-Amyl alcohol	12 h, 96.53%	[33]
Phloridzin, isoquercitrin	Palmitic acid	Novozym 435, 60 g/L	1:3	40–45 °C	Acetone	24 h (4.5 h), 93%, 24 h (4.5 h), 97%	[49]
Puerarin	Vinyl esters (C3–C8)	Whole-cell (*Aspergillus oryzae* GIM 3.4826), 50 g/L	1:30	40 °C	THF	24 h, 98%	[51]
Puerarin	Vinyl esters (C2–C12)	Novozym 435, 2 g/L	1:30	40 °C	THF	6 h, 98%	[18]
Puerarin	Vinyl propionate, vinyl hexanoate, vinyl myristate	Novozym 435, 2 g/L	1:30	50 °C	[Emim] [OAc]	6 h, 64.6%	[46]
Puerarin	Palmitic anhydride	Novozym 435, 20 g/L	1:15	40 °C	THF	3 h, 98.97%	This study

**Table 5:** Summary of log *p* values of flavonoids and flavonoid esters.

Flavonoid	Log *p* Value of Flavonoid	Flavonoid Ester	Log *p* Value of Flavonoid Ester	Ref.
Cyanidin-3-galactoside	−1.38	Cyanidin-3-O-(6″-dodecanoyl)galactoside	2.60	[57]
Isoorientin	0.21	Isoorientin laurate	5.75	[58]
		Isoorientin myristate	6.80	
		Isoorientin palmitate	7.86	
Proanthocyanidin	0.80	Proanthocyanidin palmitate	1.90	[33]
Puerarin	−0.48	Puerarin propionate	−0.03	[51]
		Puerarin butyrate	0.38	
		Puerarin pivalate	0.76	
		Puerarin hexanoate	1.20	
		Puerarin octanate	1.85	
		Puerarin laurate	2.64	
		Puerarin myristate	2.80	
Puerarin	−0.22	Puerarin palmitate	3.49	This study

## Data Availability

The data presented in this study are available on request from the corresponding author.
